# Peer review of "Overexpression of *BmJHBPd2* Repressed Silk Synthesis by Inhibiting the JH/Kr-h1 Signaling Pathway in *Bombyx mori"

_ijms, 2023, doi:10.3390/ijms241612650_

Round 1
Reviewer 1 Report
This manuscript describes about the relationship between silk protein production and JHBPd2 which discovered in the posterior silk gland (PSG) of Bombyx mori as JH binding protein. First, the authors focused on the point of the differences of JHBPd2 expression between high and low silk producing strains, and then found that the JHBPd2 was more expressed in the high silk producing strain than the low silk producing strain.
On the basis of the finding, the author performed the overexpression experiment of JHBPd2 gene using transfected cells and transgenic silkworm.
In the view of the improvement of silk production, it is remarkable that the author works regarding JHBPd2 gene function.
However, conclusionary, the overexpression of the JHBPd2 gene occurred the reduction of major silk proteins of Fib-H and Fig-L and the authors discussed the reason in the connection with JH/ 20E and their related genes.
Major questions and comments:
1. Is the JHBPd2 gene really related with the production of silk proteins? Because the first hypothesis of the authors written above is broken by the contradiction of between their hypothesis and data.
2. Although the authors describe that JHBPd2 works as keeper of JH maintenance, that is, which catches JH derived from blood in the PSG and releases it in the appropriate timing, however, the authors do not show the evidence of that interaction of JHBPd2 and JH. Also, regarding the authors’ insistence of the production of JH in PSG, they have not shown the data. I recommend examining the titer of JH and 20E.
3. I recommend the following additional works to verify the relationship of the silk production and JHBPd2 gene.
*To investigate the expression of JHBPd2 gene in the several tissues beyond PSG
*JHBPd2 gene knock-out experiment.
*Analysis of the expression of other genes, such as Bmdimm, Shade.
Other detail comments (see below),
Abstract
*Line25: *Clearly describe that “BmJHBPd2OE” expressing BmJHBPd2 means transgenic silkworm.
Introduction
*Line42: “occurs” -> “works”
*Line46: I can’t understand the phrase “which are considered juvenile hormone receptors” which does not seem to be appropriate here. Is it that “JHBP” is equal to “juvenile hormone receptors?”
*Line48: Remove the second “progress” in the sentence.
*Line49: “The expression level of JHBP was predominantly expressed in the fat body of the bamboo borer was at the medium level from the 3rd to 5th.”
-> “The expression level of JHBP which was predominant in the fat body of the bamboo borer, was at the medium from the 3rd to 5th.”
*Line66: “glycoprotein protein” -> “glycoprotein”
*Line82: “highly” -> “high”
Results
*Line93: What does “four main economic characteristics” indicate?
Although in the next sentence you describe as “The cocoon, pupae, and cocoon weight, particularly the shell weight percentage”, what is the four? Cocoon and pupae are not economic characteristic. Aren’t the “cocoon” and the “cocoon weight” used as the same mean?
*Line94: Aren’t the “cocoon” and the “cocoon weight” used as the same mean?
“(Figure S1A and B)” -> “(Figure S1B)”
*Line118: Delete the “firstly”
*Line121: “Those results are” -> “This result is”
*Line131: “the control group” -> “pSLfa1180-basic”
*Line132: “the experimental group” -> “pSLfa1180-BmJHBPd2”
*Line132: Delete the “This suggested that the overexpressed BmJHBPd2 protein reduced the response of BmE cells to JHA.”, because in the following sentence there is the similar phrase.
*Line136: “in all our experiments” -> “in all experiments using BmE cell”
*Line137: “to silk proteins” -> “to silk protein genes”
*Line154: “Transgenic” -> “The vector”
*Line155: How many positive broods did you have? Only one? If you had other line were the responses the same?
*Line157: I recommend changing “L53D” to “L5D3”. In other places as well.
*Line158: “The PSG showed” -> “The result showed”
*Line159: “than in” -> “than that in”
*Line160: “BmJHBPd2” is not a gene. It is a protein so it should not be written by Italic. Check in other places as well.
*Line161: “have shown” -> “showed”
*Line162: “in the BmJHBPd2OE strain” -> “in the transgenic line, BmJHBPd2OE”
*Line163: “have indicated” -> “indicated”
*Line217: “transcript” -> “transcription”
Discussion
*Line238: “suppressed JH signaling by inhibiting the expression of Bmkr-h1.”
You don’t show in Figure 2 the fact regarding the suppression of JH signaling.
*The discussion after L245 was mainly proceeded on the basis of the author’s inference.
Although in Line 251 the authors describe as “the 5th instar silk gland of the silkworm is likely to contain JH, and that JHBPd2 can play a role in regulating the concentration gradient of JH in the silk gland functioning”, that fact have never been shown in this study or even references.
*Line258: Regarding “Kr-h1 can directly inhibit the biosynthesis of 20E”, the story of the additional biosynthesis of 20E by the reduction of Kr-h1 is a story only in the PSG. In the Figure 5, why does the weight of pupae decrease? The size of PSG and the reduction of the cocoon shell weight dose not explain.
Also, doesn’t the increase of 20E induce the cell death program?
*Line261: ““a which” -> “which”
*Line271: “This contradictory result”
Regarding it, I think the authors can’t explain enough. Although S872 more expresses BmJHBPd2 than that of Dazao as shown in Figure 1D, why S872 have more weight silk layer than that of Dazao? That is opposite to the results in the transgenic silkworm experiments.
*Line274: “This suggested that high silk yield S872 has more JH in vivo than Dazao”
Regarding it, the authors develop the story without the evidence (not shown as data).
Also, doesn’t the BmJHBPd2 specially expressed in the PSG? Thereby, can BmJHBPd2 regulate the developmental time and body size?
You should research the other place of expression of BmJHBPd2 gene beyond PSG.
*Line275: “a which” -> “which”
*Line276: is there the evidence of “With more JH in the high silk yield S872”?
*Line281: “the amount of JH in the silkworm was not affected” is no evidence.
*Line281: “. with silk yield being a quantitative trait,” is it grammatically correct?
*Line291: “The JH in JHBPd2OE line silk glands was not measured in this study, because there was too little material available. We performed JH assays on normal silk gland tissues and found that the silk glands contained JH (data not published).
Though they did JH assay in the normal silkworm PSG, why can’t the author perform the assay of JH in the PSG of Transgenic silkworm?
*Line297: “This complex activates the expression of BmKr-h1, and subsequently, the expression of the transcription factor Bmdimm to regulate Bmfib-H”
This is the hypothesis of the authors of Reference #31. The authors in the present paper should confirm it. I recommend examining the expression of Bmdimm.
*Line297: “Cytoplasmic BmJHBPd2 can bind to redundant JH and slowly release it to maintain the JH level, which continuously regulates gene expression for silk synthesis.”
Regarding it, normal silkworm (S872 strain) expresses BmJHBPd2 at high level in the day 5th of five instar as shown in Figure 1. If the authors’ hypothesis is correct, the massive BmJHBPd2 catch the free JH, then decrease the production level of silk in the S872, even unlikely less than that of Dazao strain.
There is a critical question; does BmJHBPd2 really regulate the silk synthesis?
(Sure, though BmJHBPd2 might have interaction with the genes relating the JH/Kr-h1 signal pathway.)
Materials and Methods
*Please describe the methods understandable and adequate information for reader to reexamine. Much information is not enough such as products number, PCR condition, cDNA construction, Solution I, and so on.
* Describe what you did in order. For example, in making a plasmid, clearly state which plasmid you are making first by name and describe how you made it in detail.
*Provide a citation for any plasmids and injection method for the production of transgenic silkworm you have used previously. If you have shown the diagrams of plasmids, please add figure numbers.
*In Section 4.2, was JHA written in Figure 3 melted in DMAO? Add the information of the concentration of JHA and what kind of JHA you used.
*Line327: “the eggs”-> “the cell”?
*In Section 4.3, first start to write from the construction of the plasmids used here and understandably in order.
*In Section 4.4, add the information of the extraction way from the PSG, not only cells.
*Line349: “Western blot” -> “Western blotting”
*Line359: Move the “secondary antibody” to Line358 like “with the secondary antibody of goat anti-rabbit IgG labeled”
*In Section 4.5, I think “proportion” is appropriate in this case, not “rate”.
*Line365: “which was measured in 15 transgenic lines and wild-type individuals” might confuse readers. I think you use one line of transgenic silkworm, and each 15 individuals from transgenic and wild-type (Dazao); clearly describe wild-type means Dazao.
*Line367: “Plasmid Construction” -> “Plasmid Construction for production of transgenic silkworm”
Because you describe the plasmid construction for cell transfection in Section 4.3.
*In Section 4.8, add what kind of t-test you used. Student, Welch?
Figures
Figure 1.
*In the explanatory notes, “872” -> “S872”
*Do remove the “ns” or describe it in the caption.
*Line110: “days” -> “day”
* Line110: Remove the “as” in the.
*“BmRpl3 expression is shown as a control” -> “BmRpl3 was used as an internal control”
Figure 2.
*Unify the vector name of “pSLfa1180 [A4-GFP-JHBPd2-myc]” in the A and B, and the text (manuscript) as well.
*In the B, the GFP expression image of the pSLfa1180-GFP is completely different from that of the pSLfa1180 [A4-GFP-JHBPd2-myc]. Although those vectors use the same of A4 promoter, those are seen expressed in the nucleus and the cytoplasm, respectively. Why?
*Add a size bar to all pictures.
Figure 3.
*Line143: “protein level of in” -> “protein level in”
*In the A, I think that the explanatory notes in the upper are not necessary, which is duplicated. Instead of that I recommend adding subtitles, “qRT-PCR” and “western blotting.”
*Line146: Move the “assayed by qRT-PCR” behind the legend of D like “The experiments of B to D were assayed by qRT-PCR and BmRpl3 expression was used as an internal control.”
*To be understandable for reader, add “JHA (-)” in the C and “JHA (+)” in the D. Instead of that, remove the JSA+” from the explanatory notes in the upper subtitle of Figure 3D.
*If you have the data of BmMet-1 in the D, add that because the effect to the BmMet1 is on my mind.
Figure 4.
*In the A, redraw the vector map in more detail and correctly. Show the two “Fib-L” separately to the “Fib-L promoter” and “Fib-L terminator” and add piggyBac arms.
*Line167: I guess that “the right shows a positive individual and” is not correct. I think both photographs are transgenic, not only the right.
*Line170: “, with BmRpl3 expression being shown as a control.” -> “. BmRpl3 expression being used as an internal control.”
*Add the description of WT and OE in the capture.
*Line171: “* P < 0.05; ** P <0.01; *** P < 0.001.” -> “*** P < 0.001.”
Figure 5.
*Each picture should be enlarged, especially in the PSG.
*The captions along the vertical axis in C to E had better turn into “weight,” because of the duplication.
*D, “Cocoon weight” -> “Cocoon shell weight” or “Pupa weight”?
Because those are different from the alignment in Figure S1.
*The captions along the vertical axis in F had better turn into “proportion.”
*F, “Cocoon shall layer” -> “Cocoon shell” (spell check!)
*Add a space between C and D or E and F.
*Line198: “* P < 0.05; ** P <0.01; *** P < 0.001.” -> “** P <0.01; *** P < 0.001.”
Figure 7.
*Line230: “* P < 0.05; ** P <0.01; *** P < 0.001.” -> “** P <0.01.”
*Describe the “ns” in the caption.
Supplementary Materials
* Put the table titles over the tables.
Table S2.
*Remove the dot as the “Table S2.”
*Make the fonts unify and remove the underbar under the sequences.
* “5’-3’” -> “primer sequences (5’-3’)”
Figure S1.
*The captions along the vertical axis in B to D had better turn into “weight,” because of the duplication.
*D, “Cocoon weight” -> “Cocoon shell weight”?
*The captions along the vertical axis in E had better turn into “proportion.”
*E, “Cocoon shall layer” -> “Cocoon shell” (spell check!)
*Add a space between B and C or D and E.
Others
*Regarding the notation of “GFP,” I recommend unifying the “EGFP” in the whole places, text and figures.
*In the whole bar graph, make a distance between the vertical lines showing significance and the lines of the SD like Figure 1.
As for the quality of English, I feel that there are many places that need to be corrected in terms of grammar and expression.
I recommend hiring a company to proofread your English.
Reviewer 2 Report
Authors studied a possible role of a juvenile hormone (JH) binding protein of Bombyx mori, BmJHBPd2, for silk synthesis in the posterior silk gland (PSG) of the silkworm. Their molecular biological data on BmE cells give some evidence that JH regulates silk protein synthesis, but the results are contradictory. The Experiments were well planned but, unfortunately, authors were not able to prove any JH in the studied cells. And, as well known, lepidopterans do not have only one JH but three homologs. My most serious concern, however, is that authors do not say anything on the JH analog used in their experiments as well as on its concentration! Another serious flat is that authors used a simple t-test for the statistical analyses of their data without checking normal distribution of their values, and that with an "n" of only 3!
Some minors:
Introduction: you probably mean hemiterpenoid?
Results: data in Fig. S2 are very important for the paper and should be shown here. What do you mean with pupau and pupaus? Why is Fig. 1D shown in other colors? Please show only those significances in the figure legends that are used there.
Discussion:
Correct is corpus allatum or corpora allata. Much of the discussion is highly speculative as long as concentrations for JH I to JH III in silk glands are not known. It would be really interesting to find JH biosynthesis in silk glands, but that is again speculative.
Materials and Methods:
Give dilutions for all antibodies used.
The manuscript needs considerable language and
spelling improvement. The references must be
checked for uniform style of presentation according to MDPI Authors' Instructions.
Round 2
Reviewer 1 Report
Thank you very much for revising the manuscript, and acceptance of my comments.
First, I want to say the respects to you for the many efforts to this research.
Although I choose the major revision this time, after the nest revision this article will reach acceptable, I believe.
Yes, the target gene, BmJHBPd2, may be likely the relationship with the silk production, however, in this article there are only circumstantial evidence not direct evidence; you show the disturbing phenomenon of genes related JH/20E signal pathway, while not the direct interaction of BmJJHBPd2 and JH. Unfortunately, the hopeful result of high silk production also has not seen.
I ask you to add some following information from the cover letter to the manuscript that may help for readers to understand that BmJHBPd2 relates with the silk production.
1. To add the Re-Fig3B and Re-Table 1 including the result in JHBPd2 OE line which could not detect JH as the Supplementary Material, which should have readers image that JH in PSG were trapped by BmJHBPd2.
Since the numbers and letters in the original data is too small, the display in those should be visible in a new figure.
2. To add the Knock-out experiment of BmJHBPd2 which should show the significant decrease of silk. That would help reader understand that BmJHBPd2 related with the silk production.
I think it better to be put after Fig 1 or in the Supplementary Material.
Other comments.
(The followings are written in according with the orders in the author’s cover letter.)
#1-7:
OK
#8:
Thank you for the revising Figure 5 and Figure S1.
However, the “Whole cocoon weight” and the “Cocoon weight” very similar and each “weight” would be not necessary.
Accordingly, I recommend the followings notation.
“Whole cocoon” in Figure 5C and Figure S1B.
“Pupea” in Figure 5D and Figure S1C.
“Cocoon shell” in Figure 5E and Figure S1D.
“Cocoon shell” in Figure 5F and Figure S1E.
*Though the notations of “Cocoon shell” are duplicated, those are understandable at the sight.
#9-15:
OK
#16:
Thank you for the answer.
Please add the following to the section 2.3 (Line 153, in front of the “and used to establish the transgenic overexpression line”).
“We obtained four positive G2 generations, and the results of the investigations on all four G2 showed that the synthesis of silk proteins was affected. One G3 generation was conserved and continued to be reared and investigated, and the result still the same, which is why we were convinced that overexpression of BmJHBPd2 inhibits the synthesis of silk proteins.”
#17-19:
OK
#20:
It has not corrected yet. It is not a gene but a protein. Not italic.
“BmJHBPd2” with a Myc-tag -> “BmJHBPd2” with a Myc-tag
#21:
OK
#22: (Line159)
How is it to modify more as the following.
“The signals with the Myc antibody were only detected in the transgenic line (BmJHBPd2OE) but not in the WT line (Figure 4D).”
#23-24:
OK
#25:
This experimental data is hard in understanding because the factors of BmJHBPd2, JH, Bmkr-h1, reciprocally affects each other; in Figure 3B the increase of expression of BmJHBPd2 and Bmkr-h1 with JH subject to more make hardship to understand.
In comparison with the data of the BmJHBPd2 expression between Figure 3A and 3B, that in Figure 3A extremely show much more than that in Figure 3B.
Thereby, if it is tolerant to discuss the BmJHBPd2 expression between Figure 3A and 3B in the same place, I recommend to emphasized that pint; the excessive expression of BmJHBPd2 in the transfected cells reduced the amount of JH and then lowered the expression of Bmkr-h1, even though the expression of Bmkr-h1 in Figure 3B increased.
(#24 and 25 are duplicated)
#Second 24
See #33.
#Second 25
Regarding the “In fact, the synthesis of silk proteins and the development of the whole body is a coordinated process, and when the synthesis of silk proteins is affected, it also affects the development of the individual.”, I cannot all agree, because there are some silkworm strains which has a deficient gene related with silk production but can grew without abnormality.
If the decrease of pupae and silk shell weight seen in the transgenic line attributes to the overexpression of BmJHBPd2, you should examine.
I think there are two reasons for the decrease.
One is that the overexpressed BmJHBPd2 secrets from PSG cells to blood side and then affects to body size and so on. (Does BmJHBPd2 have a secretion signal?)
Second is that there might have been some matters in the experiments such as rearing, feeding, Illness
In the first case, you may find whether BmJHBPd2 affects in outer of silk gland by an experiment of western blotting with the Myc tag antibody using the transgenic silkworm.
In the second case, you need to examine it by rearing again.
In any cases, you should give in answers to readers.
#26:
OK
#27:
My questions almost cleared.
Also see #33.
#28:
It is okay.
Simply I have a question whether there is a strain with the same developing timing but with different silk productivity. If there is, it would be a good sample.
#29:
Please more revise in Line 268 as the following,
“provides further evidence” -> “came to the idea of”
You should pay attention to use separately the definitive or euphemistic expression in accordance with whether there is the clear evidence or not.
Please check in other places as well.
#30-32:
OK
#33:
Thank you for the detail explanation. I was almost convinced.
However, in the hypothesis of catch and release of JH by BmJHBPd2 I cannot agree at least its release. You should change the definitive expression to euphemistic because the direct evidence of the catch and release is not verified at all, indeed though there are some circumstantial evidences regarding the catch.
You should clearly write that the issue of the catch and release have remained in future research.
#34:
OK
#35:
Thank you for the revision.
However, add the construction process of pSLfa1180[A4-EGFP-BmJHBPd2-SV40]. (Refer to the ref. 31: p974)
#36:
OK
#37:
Thank you for the revising.
Please add “JHA was melted in DMSO, was from the reference (31? Zhao et. al, 2015) in the section 4.9.
#38-42:
OK
#43: (Line 369)
How do you think to modify as the following.
“which was measured in each 15 individuals of the transgenic and wild type silkworms of Dazao."
#44-51:
OK
#52:
It is okay.
I misunderstood that the pSLfa1180-EGFP has A4 promoter. I am sorry.
EGFP image in the pSLfa1180-EGFP is non-specific, right?
#53:
Thank you for the revising. I think that the numbers of 40 are not necessary in all the pictures. That’s okay, only in the left bottom of picture.
#54-58:
OK
#59:
Thank you for the revising. I think it better to add piggyBac arms (ITR).
#60-64:
OK
#65-69:
OK
#70:
In Table S2, the description beyond of title should be put under the table.
#71:
OK
#72:
See #8.
#73:
OK
#74:
The new figure, Figure S2, is not corrected yet. The others are okay.
Additional suggestions.
#75: (In the section 4.6)
Add the truncated fragment region after BmJHBPd2 as the following, “BmJHBPd2 (-XXX to +XX).
#76: (In the section 4.6)
Which do you yourself made the cDNA (Line375) or not?
If you made it, you should add the process of making (refer to the ref. 22), but if you receipt from others, you should add it in the acknowledgement.
#77: (In Table S2)
I think it better to modify seeing the Table 3 in the ref. 31.
#78: (In Figure S1)
“872” -> “S872”
There’re in five places.
Modify as the following, “Main characteristics of different silk-producing strains of Dazao and S872”
In the legend, remove the “from Dazao and S872” in A to D, and In B, “Cocoon” -> “whole cocoon”
I recommend checking and improving again.
Reviewer 2 Report
Authors have revised the manuscript according to my suggestions, but it still needs some additions and corrections.
Please add your proof of JH and 20E in the silk gland as a figure (LC-MS) or table to the Supplementary Material. To mention JHA from Sigma is not enough. The correct name of the supplier is Sigma-Aldrich and you must say which JHA was used (methoprene, catalog number?). References still do not match the specifications of MDPI Authors' Instructions (journal names).
Some sentences are not complete.
Round 3
Reviewer 1 Report
Dear authors,
Thank you for the second revision.
I attached my comments below.
Major comments:
I think that it is important to show the data of JH amounts differences in PSG of the transgenic silkworm and WT, and of Knock-out experiment of BmJHBPd2; which may make readers convinced that BmJHBPd2 is related with the silk production through JH.
However, unfortunately, those have not been shown in this version.
Therefore, I asked the IJBS editor to give you the time enough to take those data and leaved to him the decision of whether accepting this article this time.
Minor comments:
#8:
Thank you for revising Figure S1.
However, it was not done yet in Figure 5 of the manuscript, though it was already corrected in your letter.
#16: (from Line 153 to 158)
Thank you for revising.
I modified a little to make the text flow better as following.
“An EGFP-positive brood was obtained and used to establish the transgenic overexpression line (Figure 4B). Then we obtained four positive G2 generations, and the results of the investigations on all four G2 showed that the synthesis of silk proteins was affected. One G3 generation was conserved and continued to be reared and investigated, and the result still was the same.”
#20:
OK
#22:
OK
#25: (to Line 131)
Thank you for revising.
I added a little to make understandable for readers as following.
“…….. the expression of Bmkr-h1, since the overexpression of BMJHBPd2 may be high enough as shown in Figure 3A, even though the expression of Bmkr-h1 in Figure 3B increased.”
#Second 25:
Please add references regarding the “In fact, the synthesis of silk proteins and the development of the whole body is a coordinated process, and when the synthesis of silk proteins is affected, it also affects the development of the individual.”
Many readers would want to know why.
Even though hypothesis, abnormality of silk production might make silkworm metabolism worse and then the body size would have lowered because there is likely that silkworm discharge the waste for silk outside of the body. However, while the naked pupae with failed silk production sometimes have heavy weight.
#28:
OK
#29:
OK
#33:
OK
#35:
I didn’t see the difference.
Please rewrite the construction way of pSLfa1180[A4-EGFP-BmJHBPd2-SV40] seeing the ref. 31: p974, and pSLfa1180-EGFP as well.
Especially, I thought that “pSLfa1180-EGFP” did not have A4 promoter but yes does (See #52).
#37:
OK
#43:
OK
#52:
If “pSLfa1180-EGFP” has A4 promoter, I look the image of EGFP field wrong because it should show the expression in the cytoplasm but not in the nuclei.
How do you think?
#53:
Thank you for revising.
Because of #52, I think it better that “pSLfa1180-EGFP” should turn into “pSLfa1180[A4-EGFP]”.
(Please check the whole manuscript.)
#59:
OK
#70:
OK
#74:
OK
Thank you for revising Figure S2.
However, it was not done yet in the manuscript, though it was already corrected in your letter.
#75:
I didn’t see the difference.
Please check it.
#76:
It’s okay. I mistook. I am sorry.
#77:
OK
#78:
“872” -> “S872”
It has not corrected yet.
(Five places)
Sincerely
I hope to be made it better, especially in grammar.
Round 4
Reviewer 1 Report
Thank you for revising the manuscript.
I checked and saw leaving little to be desired excluding the major I previously pointed out and #52.
Finally, I will leave to the editor the estimation of both issues.
Sincerely.
Minor:
#8:
OK
#16:
OK
#25:
OK
#Second 25:
Thank you for replying of your sincerely answer and thought. It is interesting thing whether the phenomenon in PSG affects to the development of the worm such as body weight and size. I expect your future work.
#35
OK
#52:
I can’t understand this phenomenon only in the information you describe because I recognize the A4 promoter express in the cytoplasm. Does the EGFP have nuclear localization signal? Also, I have not heard that the expression of fusion proteins with EGFP were stronger than the original EGFP. As a reason as possible as I think, although EGSP has nuclear localization signal, the fusion protein with BmJHBPd2 move from nucleus to cytoplasm by the function of BmJHBPd2 which strongly transfers to cytoplasm. How is it in fact? Please give the convinced answer.
#53:
OK
#75:
OK
I agree with you.
#78:
OK
Please try to further elaborate.