# Supplementary material for: Overexpression of BmJHBPd2 Repressed Silk Synthesis by Inhibiting the JH/Kr-h1 Signaling Pathway in Bombyx mori
Source: Int J Mol Sci. 2023 Aug 10;24(16):12650. doi: 10.3390/ijms241612650 (PMC10454397; doi:10.3390/ijms241612650)
Supplement: Supplementary file 1 [file ijms-24-12650-s001.zip › ijms-2441055-supplementary.pdf]

Supplementary Materials

Table S1. Primer sets used in the transgenic overexpression.

Table S2. Primer sequences used in this study.

Figure S1. Main characteristics of different silk-producing strains of *Dazao* and *S872.*

Figure S2. Overexpression of *BmJHBPd2* results in inhibition of JH catabolic enzyme in silk glands.

Table S1. Primer sets used in the transgenic overexpression.

Gene	Primer sequences (5′-3′)
BmJHBPd2-F (*BamH* I)	GGATCCATGTGGACCGGTCTGTTTTTAGT
BmJHBPd2-R (*Not* I-Myc)	GCGGCCGCTTACAGATCCTCTTCTGAGATGAGTTTTTGTTCTTCGGGCATTAATTCGTCAA

The underlined sequence is *BamH* I restriction site, *Not* I restriction site and Myc tag sequence.

Table S2. Primer sequences used in this study.

Assay	Gene		Primer sequences (5′-3′)
RT-PCR	*BmJHBPd2*	F,	ATGTGGACCGGTCTGTTTTTAGT
		R,	TTCGGGCATTAATTCGTCAA
RT-PCR	*BmRPL3-1*	F,	TCGTCATCGTGGTAAGGTCAA
		R,	TTTGTATCCTTTGCCCTTGGT
qRT-PCR	*BmJHBPd2*	F,	TAGAAAAGGCTGTGTTTCGC
		R,	ATGAGTGATCCGCAGATTT
qRT-PCR	*BmfibH*	F,	TCTGTGTCATCTGCTTCATCTCG
		R,	TATCCAGGACGAAGTAAGAAACAA
qRT-PCR	*BmfibL*	F,	ATACCGATTGGTCACATAACAG
		R,	GCAGATAGATGGGCGATAA
qRT-PCR	*BmP25*	F,	AGCCGCTGTGGCAGTTTTG
		R,	TAGGTGGCGTTGAAGTATGG
qRT-PCR	*BmKr-h1*	F,	CTTCCTCCTACTCCACCT
		R,	GGCAACGAAATGTAATGT

qRT-PCR	*BmMet1*	F,	AATCTTGCCACCAACAGC
		R,	ACCCAACGCACATCTTCT
qRT-PCR	*BmMet2*	F,	CCGAACCAACGCAGTATGTAA
		R,	ACGCACGACGCCAATGA
qRT-PCR	*BmSRC*	F,	TCAAACGAGTCAAATAGGGTCA
		R,	GCGGTCGGTGGTAGGGTT
qRT-PCR	*Bmjhe*	F,	ACAGATTGGCGGTTTTCGGA
		R,	CCCTTAGCGGCATCAGACAT
qRT-PCR	*Bmjheh*	F,	GGACTCTTCGGGTTAGTGATAA
		R,	TGTGGTATTTCGTGCATTCC
qRT-PCR	*Bmdimm*	F,	CGTGGAACCCGCATTTGTA
		R,	AACCTCGGCAATCCAGTCG
qRT-PCR	*Bmsage*	F,	AGCAATCACGAAGGTCCGC
		R,	CGTATCGTGGTTGGAGTCGT
qRT-PCR	*Bmsgf-1*	F,	ATCCGACATTCGCTGTCCTT
		R,	TGACGTCGCAAGAAACAACC
qRT-PCR	*BmEcR*	F,	GCTGGTCTGATAACGGTGGCT
		R,	CAAGGATTCCGGCGACATAAC
qRT-PCR	*BmHR3*	F,	TCAACGAGAAGACACCACGG
		R,	AGAAACATCCTGGGGCTTGC
qRT-PCR	*BmE75A*	F,	GAAATTCCGCGCTATGAGGC
		R,	TTTTAGCGAGCACCGAATGC
qRT-PCR	*BmE74A*	F,	AGCAGTCAACTGCAAGGGTA
		R,	GTGCCCGATCTAAGGAGTTG
qRT-PCR	*BmUSP*	F,	AGAAGTGGACGTTCTTCGAG
		R,	GGTGGAACAGGTAGAGGTGC

qRT-PCR	*BmBrc*	F,	TCGCTGACAAACACGCTG
		R,	ATGGTAAGAACGGCGGAC
qRT-PCR	*BmRPL3-2*	F,	TTCGTACTGGCTCTTCTCGT
		R,	CAAAGTTGATAGCAATTCCCT

F indicates forward, and R indicates reverse.

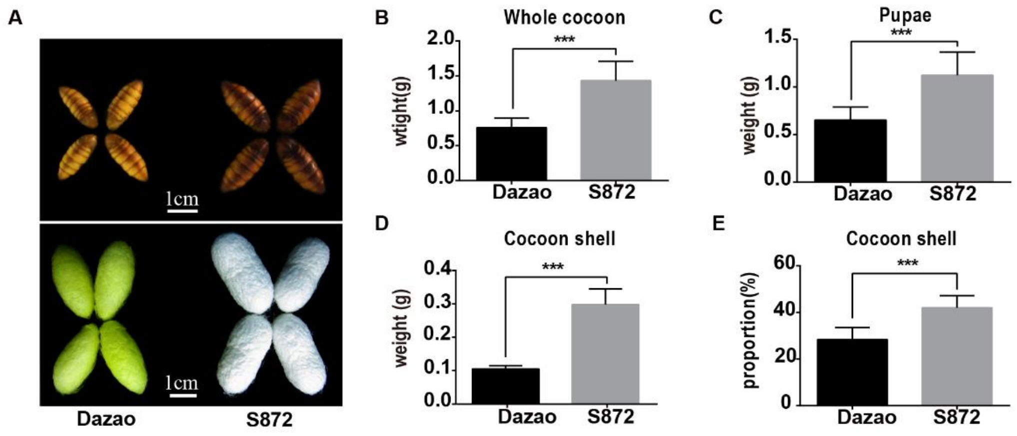

Figure S1. Main characteristics of different silk-producing strains of *Dazao* and *S872*. A. Pupae and cocoons. B. whole cocoon weight. C. Pupae weight. D. Cocoon shell weight. E. Cocoon shell proportion. Data are means ± SD. ***$P < 0.001$.

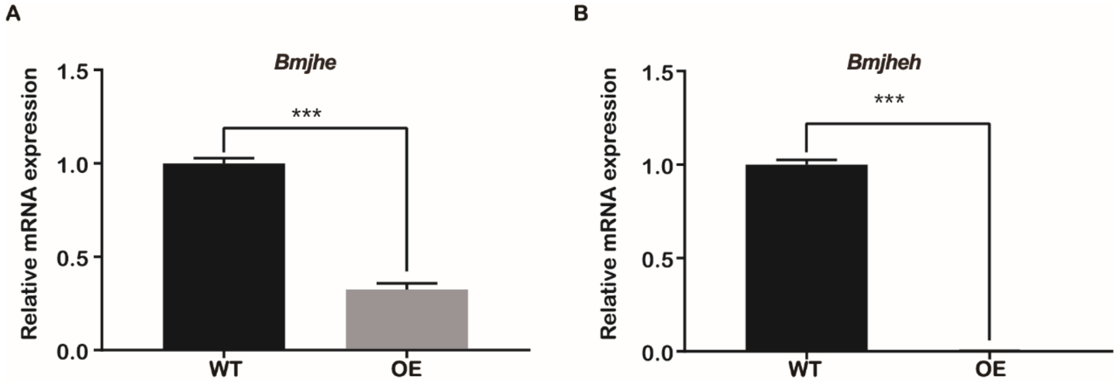

Figure S2. Overexpression of *BmJHBPd2* results in inhibition of JH catabolic enzyme in silk glands. The following JH catabolic enzyme related genes were selected: JHE(A) and JHEH(B). *BmRpl3* was used as a control. Results are expressed as means S.D. of three independent experiments. *** $P < 0.001$.
